# Interpretable data-driven discovery of high-performance multielement metal oxide electrocatalysts for oxygen evolution reaction from a small hybrid dataset

**DOI:** 10.1080/14686996.2026.2713992

**Published:** 2026-08-07

**Authors:** Wenqin Peng, Shigenobu Hayashi, Abraham Castro Garcia, Lei Fang, Masaki Takeguchi, Yibin Xu, Ken Sakaushi

**Affiliations:** a Research Center for Energy and Environmental Materials, National Institute for Materials Science, Tsukuba, Japan; b Center for Basic Research on Materials, National Institute for Materials Science, Tsukuba, Japan; c Churchill College, Cambridge, UK; d Cavendish Laboratory, University of Cambridge, Cambridge, UK

**Keywords:** Data-driven material discovery, small data, electrocatalysts, interpretable data-driven approach, multielement metal oxide, oxygen evolution reaction

## Abstract

One of the most formidable challenges in materials chemistry is the rational design of functionalities capable of dramatically enhancing performance. However, it is well-known that the discovery of promising materials often requires several decades of continuous trial-and-error. Herein, we show an interpretable data-driven framework for the discovery of multielement metal oxide oxygen evolution reaction (OER) electrocatalysts in alkaline media within a substantially shorter timeframe. This framework was trained on a hybrid dataset comprising only 557 data, consisting of the curated literature and our own experimental results. Furthermore, this framework was specifically designed to enable extrapolative materials discovery, including the exploration of elemental combinations absent from the training database. Consequently, from a large material search space of approximately 3 million candidates, we identified a promising unconventional quinary oxide composed of V, Ni, W, Rh, and Ru that exhibits, in 0.1 M KOH, an exchange current density approximately 20 times higher than that of IrO_2_. This work serves as a proof-of-concept, demonstrating that the rational design of high-performance electrochemical functionalities from an extensive candidate space can be achieved using a small hybrid dataset combined with an interpretable data-driven approach.

## IMPACT STATEMENT

This work develops an extrapolative-sniping artificial intelligence framework based on a small hybrid database comprising 557 data, accelerating the discovery of multielement metal oxide electrocatalysts with excellent oxygen evolution performance.

## GRAPHICAL ABSTRACT

**Figure:**
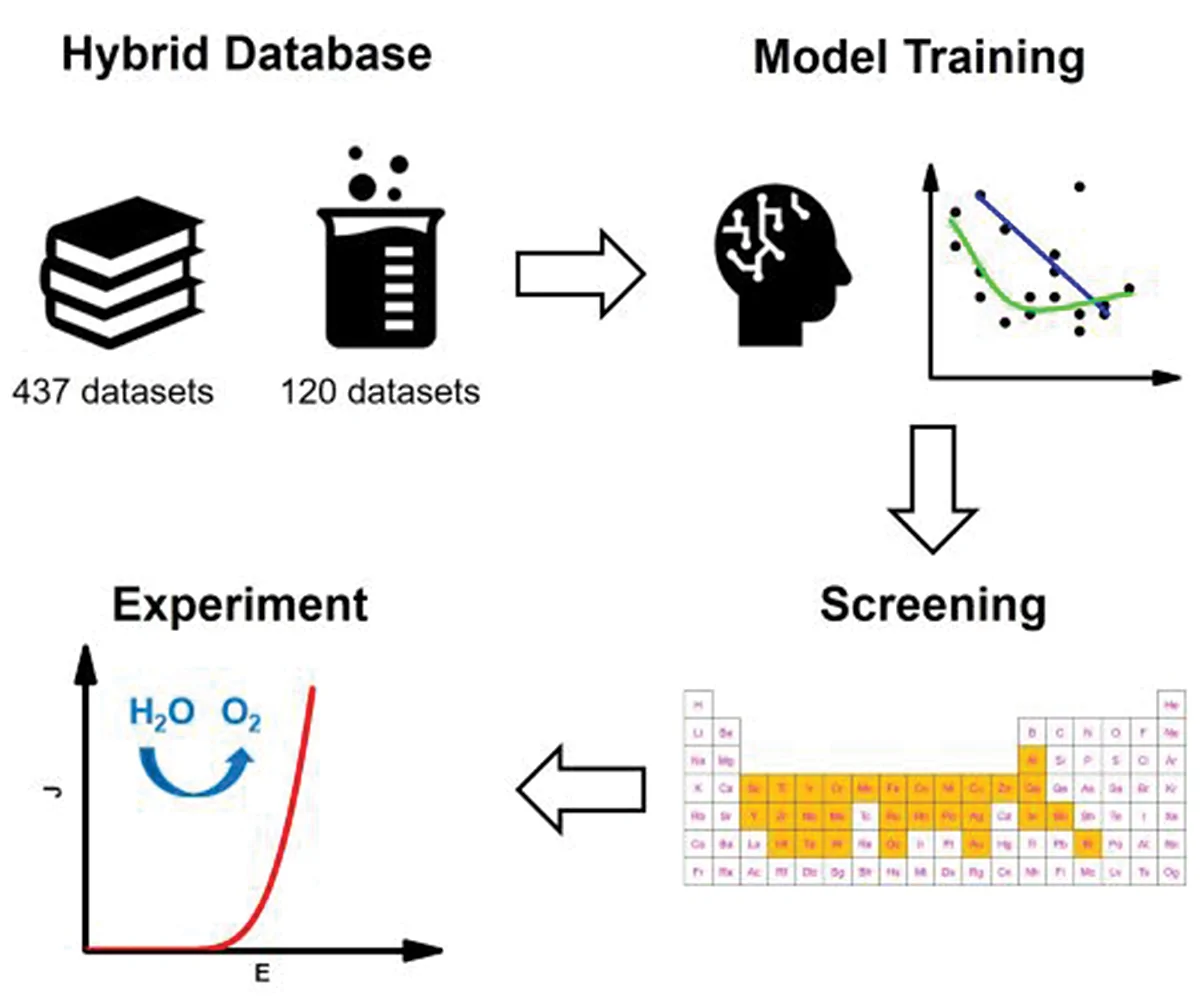

## Introduction

Finding a high-performance functionality with affordable element demands an extraordinary effort even for a single technology: Paul Alwin Mittasch, a co-worker of Fritz Haber, had attempted several tens of thousands of systematic experiments to check multicomponent material candidates combining 54 elements towards the development of the Haber-Bosch process [1,2]. The motivation for this hectic mission is not only for discovering a cost-effective catalyst with better activity, but also to understand why the specific iron oxide from Gällivare, Sweden, is able to act as a good catalyst for this process but not iron oxide from other regions. Thus, systematic experiments with large material search space are indispensable to find advanced materials towards developing modern technology and to unveil basic mechanisms of complicated chemical reactions or building up design principles to synthesize materials with desirable functions. From this background, it is of great interest to establish effective methodology equipped with artificial intelligence (AI) to shorten the trial-and-error timeframe during systematic experiments to find a material with target properties [3–6]. In particular, this methodology is important for the development of advanced electrochemical devices such as water electrolyzers, a critical climate technology for the green hydrogen production. This is because of the urgent demand for high-performance electrocatalysts for oxygen evolution reaction (OER) to meet carbon neutrality goals by 2050 [7], however discovery of promising electrochemical materials often requires several decades of continuous efforts due to complexity of electrode processes [8–11]. Moreover, multielement materials are attracting great interest due to their enhanced properties originating from non-trivial element combining effects [12–16]. However, optimization of this type of advanced materials is even more challenging compared to traditional materials. Aiming to develop high-performance multielement OER electrocatalysts, Robotics-AI combination is known to be capable of dealing with a larger search space containing millions or more of candidates, as demonstrated by its overwhelming power in several fields of materials discovery, for instance drug discovery [17–21]. However in the field of electrochemical material discovery, the unique features of electrochemical experiments cause the robotic experiments to require a long and expensive period to establish an optimized protocol to provide accurate and reliable data [22]. Moreover, a promising alternative approach of Human-AI collaboration has been proven to work however only in a candidate search space with several thousands of candidates so far [22,23]. From this point, a material prediction AI is an alternative to robotics-AI or human-AI approaches for a material discovery from a large search space.

**Figure 1.**
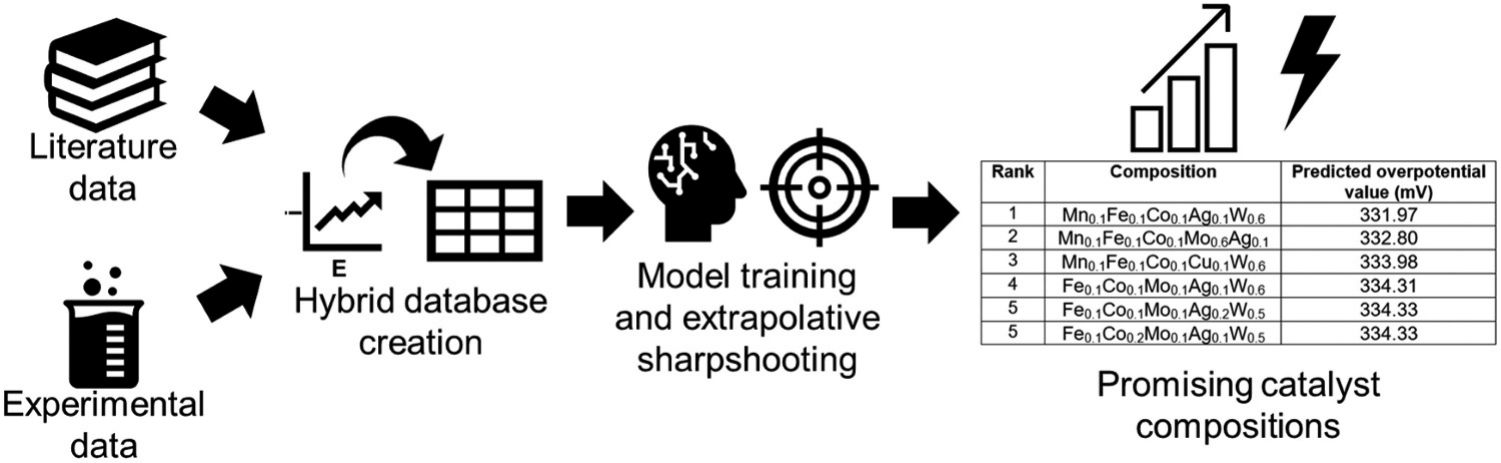
Overview of applied data‑driven electrocatalyst search approach.

Here, we have developed an interpretable data-driven framework for the discovery of high-performance multielement metal oxide electrocatalysts toward the alkaline oxygen evolution reaction from a large material search space using a small hybrid dataset. A data-driven approach with interpretability and/or explainability is essential for ‘opening’ black-boxes of present standard AI-driven material discovery [24–26]. Our approach stands on the shoulders of past and present researchers: we curated only 437 data points of transition metal oxide-based oxygen evolution electrocatalysts from the literature and integrated these curated datasets with 120 datasets derived from our own experiments to construct a hybrid database. This small hybrid database was employed to discover multielement materials using an extrapolative material prediction algorithm with 35 parameters, navigating a large search space of approximately 3 million candidates, including elements not presented in our original database. As a result, we identified a promising oxide composed of metal elements of V, Ni, W, Rh, and Ru that delivers excellent oxygen evolution performance, which was validated through experimental measurements. This work serves as a proof-of-concept, demonstrating that a promising material discovery from a large search space is able to be achieved by using a small hybrid data with datasets in the order of 10^2^, combined with a highly accurate and reliable algorithm with 35 parameters (Figure 1).

## Results and discussion

To develop the hybrid database, papers from the existing literature were collected based on expert-judgement. Our search for relevant literature resulted in 203 papers, which were then systematically analyzed, extracting the data describing experimental conditions and electrocatalyst composition. This was done by digitizing or extrapolating from graphs as necessary to match overpotential at 1 mA/cm^2^_geo_ with electrocatalyst composition and electrolyte pH, ensuring that all data is on a standardized scale for fair comparison. This resulted in a literature-based data set consisting of 437 data points comprised entirely of oxide-based OER electrocatalysts. The composition of the electrocatalysts was represented using 17 group coefficients (belonging to groups 1 to 17 of the periodic table) and 17 group weighted properties, for a total of 35 features, including electrolyte pH [27]. The calculation of these group coefficients and group weighted properties is explained in detail in the Experimental Section. Having finished curating this literature data set an additional 120 datapoints from our own previous experiments controlled under the identical conditions were added to create a hybrid database. The literature dataset covered a broad range of reported compositions and included many high-performance catalysts, whereas the systematically acquired in-house dataset supplied internally consistent data and a substantial number of relatively high-overpotential materials that are underrepresented in the literature. The two datasets provided complementary information since the systematic experimental dataset partially compensates for the heterogeneity and publication bias of the literature dataset, while the literature dataset expands the compositional coverage beyond that of the in-house experiments.

Machine learning models: Gaussian Process regression, with radial basis function kernel (GPR (rbf)), Matern kernel (GPR (mt)) and rational quadratic kernel (GPR (rq)), Natural Gradient Boosting (NGB), Random Forest (RF) and Extreme Gradient Boosting (XGB) were trained on the literature dataset, experimental dataset and finally the combined, hybrid database. The performance of the models shows that the combination of both literature and data from our own experiments yielded the best model performance as can be observed in Table 1.

**Table 1.** Accuracy (R^2^) of ML models for overpotential @ 1 mA/cm^2^_geo._.

	GPR (rbf)	GPR (rq)	NGB	RF	XGB
**Using experimental data (120 data points)**					
Train	0.865	0.885	0.996	0.963	0.934
Test	0.635	0.632	0.61	0.579	0.621
**Using literature data (437 data points)**					
Train	0.668	0.924	0.78	0.885	0.885
Test	0.437	0.453	0.579	0.595	0.581
**Using hybrid database (557 data points)**					
Train	0.946	0.967	0.971	0.982	0.949
Test	0.822	0.819	0.853	0.84	0.877

**Figure 2.**
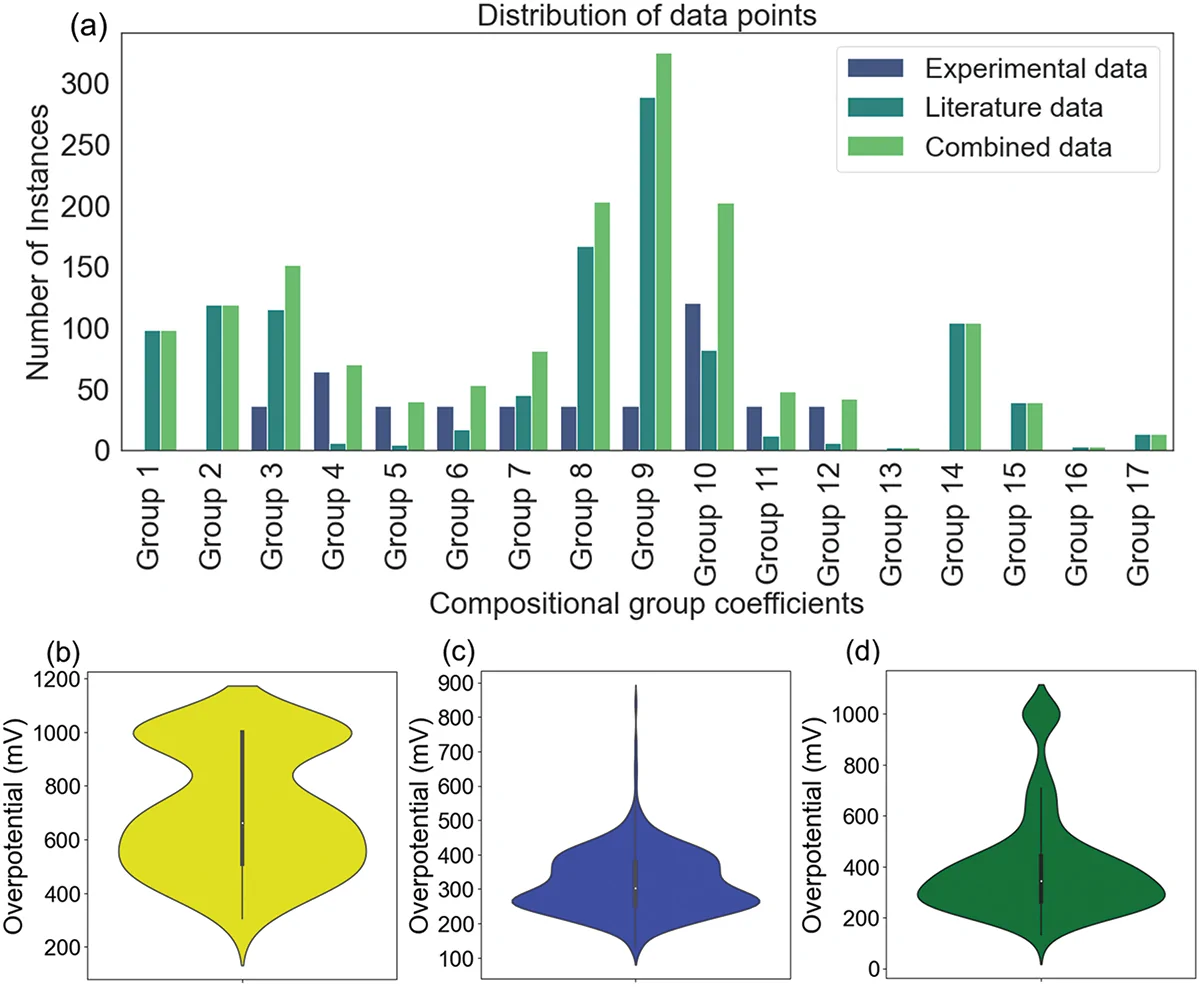
Analysis of data distribution in experimental, literature and combined data sets. (a) Bar plot comparatively showing the number of data points in each respective data set for all compositional group coefficients. (b)-(d) Violin plots showing the distribution of data points for experimental, literature and combined data sets.

To investigate why using the combined dataset resulted in significantly improved model performance, the various data sets; experimental, literature and their combination were analyzed. In Figure 2(a), it can be observed that for Groups 4, 5, 6, 10, 11 and 12, experimental data constitutes most of the data points present in the combined data set. In Figure 2(b), the distribution of overpotentials observed in the experimental data set indicates the presence of many high overpotential electrocatalysts (600–1000 mV), in contrast to the distribution from the literature data set observed in Figure 2(c), which shows an abundance of low overpotential electrocatalysts centered around 200–300 mV. Figure 2(d) shows the distribution that results from the combination of experimental and literature datasets. This combined data set is more robust, containing various examples of high and low overpotential catalysts with diverse elemental compositions. This matches well with both older [28,29] and newer [30] perspectives, pointing out that quality and diversity in data is important. The presence of ‘bad’ electrocatalysts in the dataset is crucial to enhance the likelihood of efficiently finding new promising electrocatalyst compositions.

Of the ML models trained XGB showed the best performance, although it is generally considered to be a ‘black box’ due to its lack of immediate interpretability, we decided to compute SHAP (SHapley Additive exPlanations) values to better understand the logic through which the model predicts overpotential of a given electrocatalyst as a function of the features available in the dataset. SHAP values can be used as a method to interpret ML models, although originally come from cooperative game theory. A more detailed explanation of the application of SHAP values in ML is available from Benedek, et al.[31]

**Figure 3.**
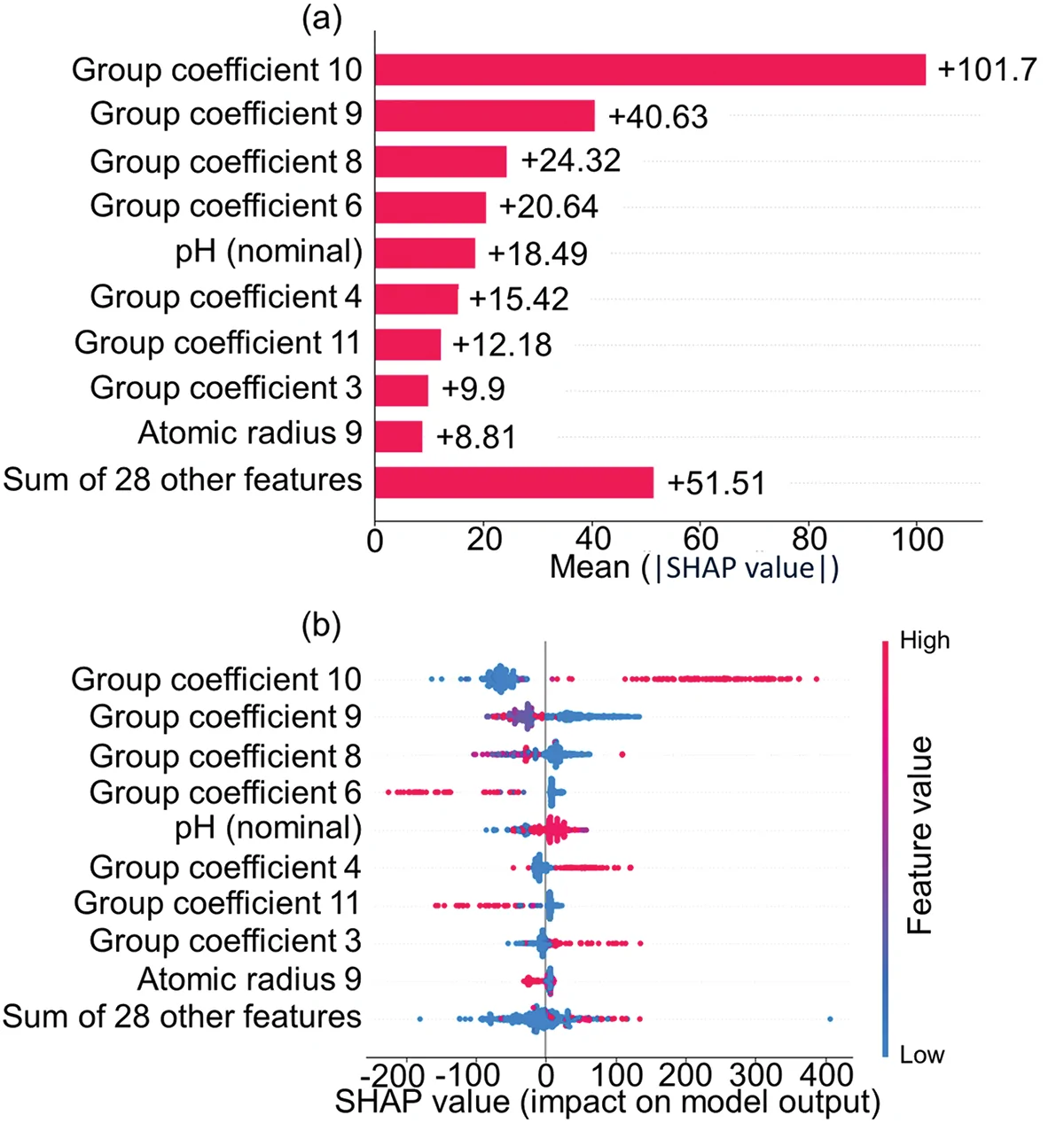
Different representations of SHAP values computed for XGB model trained using the combined dataset. (a) SHAP bar plot showing the importance of most important features. (b) SHAP bee swarm plot displaying the distribution of impact of each feature.

In Figure 3(a), the SHAP bar plot shows that only a few of the 35 available features in the data have high importance, notably, Group coefficients 10, 9, 8, 6 and pH (nominal) hold high importance. The aggregate bottom 28 features have a comparatively smaller importance, indicating that the predictions depend mostly on a few groups of elements. In Figure 3(b), the SHAP bee swarm plot, where we can observe that the absence (blue) or presence (purple to red) of certain elements is correlated with electrocatalyst overpotential. For group coefficient 10, a low value (blue, indicating the absence of elements from this group) seems to be correlated with lower overpotential, meanwhile group coefficients 9, 8 and 6 show that higher values (purple, red) show a correlation with low overpotential, indicating that their presence improves the performance of the electrocatalyst.

Previous explainable-AI studies have shown that SHAP analysis is effective for identifying composition–activity relationships in predefined multielement OER catalyst systems. For example, SHAP has been used to quantify the individual and interactive effects of Ni, Fe, Co, and Ce in (Ni–Fe–Co–Ce)O_x_ catalysts, and more recently to interpret the optimization of elemental ratios in Fe–Co–Cr–Mn–Cu high-entropy layered double hydroxides [32,33]. In these studies, the elemental candidates were fixed and the principal objective was to optimize their relative compositions. In contrast, the present model represents a broader range of oxide catalysts using periodic-table group descriptors. The resulting SHAP analysis therefore identifies compositional trends across different catalyst families rather than within a single predefined chemical system. The importance of Groups 8, 9, and 10 is broadly consistent with the central role of late transition metals frequently observed in active OER catalysts, while the identified contribution of Group 6 suggests that elements from this group may provide an additional compositional degree of freedom in multielement catalyst design. The importance of pH further demonstrates that the model captures not only catalyst composition but also the influence of the electrochemical environment.

Based on the satisfactory ML model performance achieved with the hybrid dataset and our understanding of it, an extrapolative screening approach was used to suggest new quinary oxide electrocatalyst compositions, looking to minimize overpotential at 1 mA/cm^2^_geo_. This approach considered 28 elements of the periodic table and assumed that the catalyst formula would be E1_x1_E2_x2_E3_x3_E4_x4_E5_x5_, where E stands for a given element and x_i_ can take values of 0.5 or 1. This resulted in a potential experimental space consisting of 3.04 million possible electrocatalyst compositions. The 5 trained ML models were used to calculate the overpotential @ 1 mA/cm^2^_geo_ for all 3.04 million candidates, of which the top 3000 candidates with the lowest calculated overpotential were tabulated and ranked as follows. Primary ranking: rank the candidates by the number of models recommending it, a larger number corresponding to a higher rank and secondary ranking: for candidates recommended by 2 models, further rank them by the average rank of the two models. The presumption behind this approach is that we do not trust any model more than others, given that they all achieved acceptable predictive performance. Subsequently, the most promising candidates calculated and ranked were synthesized and tested.

**Figure 4.**
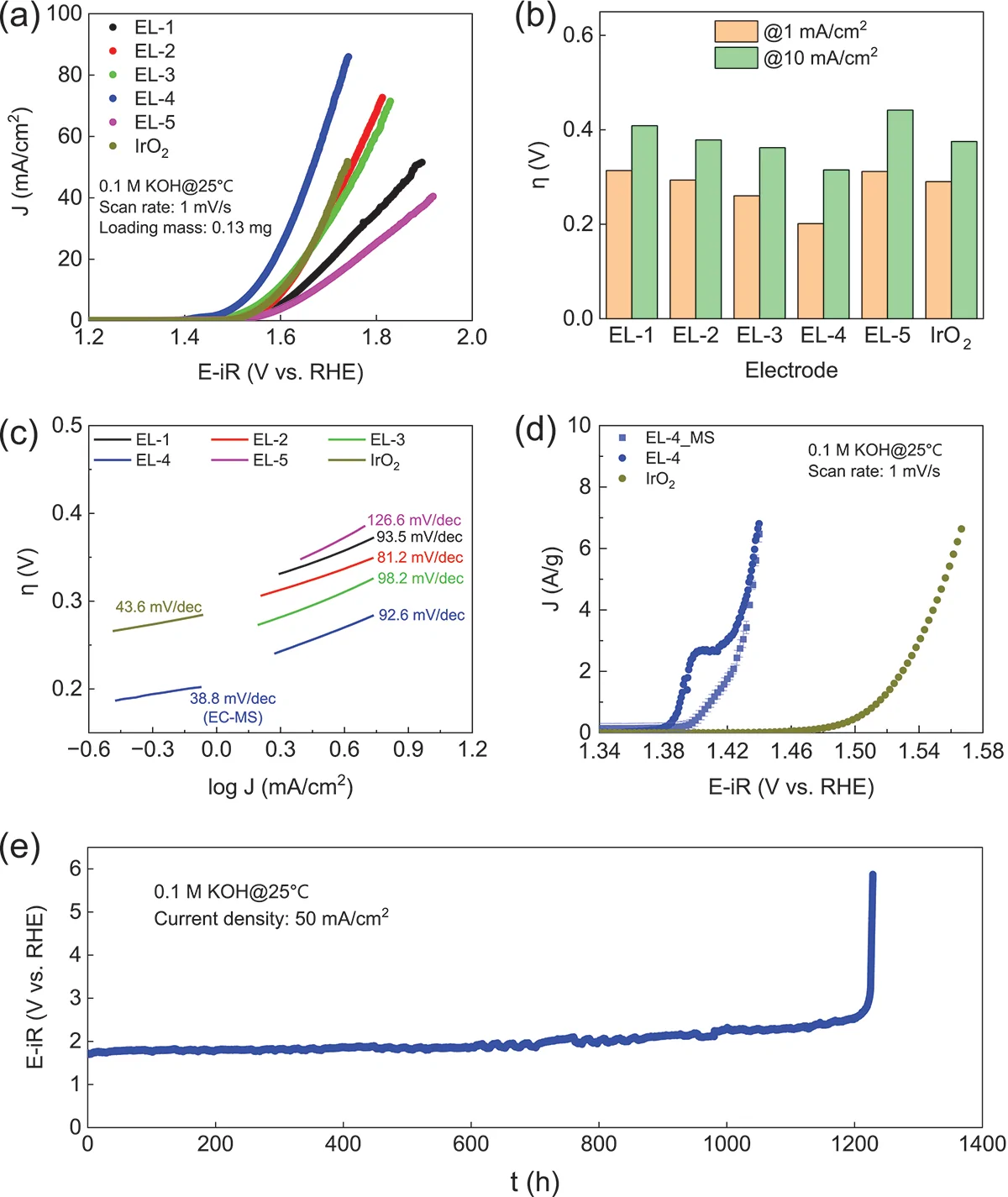
OER performance of quinary-metal-based electrocatalysts measured in 0.1 M KOH electrolyte. (a) LSV curves measured at a scan rate of 1 mV/s. (b) Overpotentials at current densities of 1 and 10 mA/cm^2^. (c) Corresponding Tafel plots. (d) Specific mass activities of EL-4 and IrO_2_ at the low potential region. Due to the redox peak, a real-time monitoring electrochemical mass-spectroscopy (EC-MS) was employed to obtain the data (square: OER oxygen current based on EC-MS measurement of EL-4). (e) Long-term durability test of EL-4 at a current density of 50 mA/cm^2^.

The five quinary-metal oxide-based compounds (listed in Table S1), which were predicted as top-ranked materials by our AI-based approach, were synthesized using a dip-coating method. The OER activities of the five materials were investigated in a three-electrode two-compartment cell configuration in the 0.1 M KOH electrolyte (pH13). We note here that the mass-loading for all the electrocatalysts is 0.13 mg. Figure 4(a) shows the *iR*-corrected linear sweeping voltammetry (LSV) curves for OER. The materials with metal element composition ratio of Ru:Rh:V:Ni:W = 0.5:0.5:0.5:0.5:1 (the Ru_0.5_Rh_0.5_V_0.5_Ni_0.5_W oxide; denoted as EL-4) were found to exhibit the best OER activity among these five electrocatalysts, achieving a current density of 81.1 mA/cm^2^ at overpotential of 0.5 V, higher than that of the in-house-prepared benchmark IrO_2_ (48.3 mA/cm^2^ at overpotential of 0.5 V). Furthermore, EL-4 shows the lowest overpotentials of 201.5 mV at a current density of 1 mA/cm^2^ and of 315.2 mV at 10 mA/cm^2^ (Figure 4(b)). The overpotential values at 10 mA/cm^2^ are 378.6 mV and 362.3 mV for EL-2 (Rh_0.5_Os_0.5_V_0.5_Ni_0.5_W) and EL-3 (Ru_0.5_Rh_0.5_Ni_0.5_Nb_0.5_W), respectively, which are comparable to that of IrO_2_ (375.4 mV), while it increases to 408.8 mV for EL-1 (Rh_0.5_Os_0.5_Ni_0.5_Nb_0.5_W). However, EL-5 (Ru_0.5_Pd_0.5_Os_0.5_Nb_0.5_W), in which Rh is absent, has a relatively higher overpotential of 441.8 mV at 10 mA/cm^2^.

As for a multistep electrochemical reaction of OER, the Tafel slope suggests information about reaction kinetics. Here the Tafel slopes were derived from their Tafel plots shown in Figure 4(c). EL-4 exhibits a smaller Tafel slope of 92.6 mV/dec, with respect to EL-1 (93.5 mV/dec), EL-3 (98.2 mV/dec) and EL-5 (126.6 mV/dec), suggesting a similar kinetics in OER except EL-5. Furthermore, mass activity (normalized by the mass loading of the electrocatalyst) is used to reveal intrinsic activity of electrocatalysts (Figure S1). At an overpotential of 300 mV, the mass activity of EL-4 is 30.3 A/g, which is more than 2 fold higher than that of EL-1 (2.5 A/g), EL-2 (5.2 A/g), EL-3 (12.3 A/g) and EL-5 (2.9 A/g). The chronopotentiometric measurement was carried out to test the durability of these electrocatalysts at a constant current density of 50 mA/cm^2^ for 3 h. As shown in Figure S2, these materials exhibit comparable durability with respect to IrO_2_ in alkaline electrolyte. Additionally, the long-term durability of EL-4 was further investigated. At the current density of 50 mA/cm^2^, EL-4 exhibits stable water oxidation operation over 1000 h (Figure 4(e)), suggesting the promising properties of EL-4. The activity at low potentials, e.g. regions close to onset potentials, is especially crucial for evaluating electrocatalysts. However, the behaviour at the low potential region is inaccessible due to the redox peak at 1.38 ~ 1.41 V vs RHE overlapping with OER response (Figure 4(d)) and moreover the oxygen produced is difficult to determine [34,35]. Therefore, the real-time monitoring electrochemical mass spectrometry (EC-MS) was employed to investigate its electrocatalytic activity within a low potential region. The mass-specific current density was calculated by normalizing the detected oxygen mass current (m/z = 32) corresponding to OER based on the mass loading of the electrocatalyst. Figure 4(d) shows that oxygen started to evolve at ~1.39 V vs RHE, reaching a specific current of 6.5 A/g at 1.44 V vs RHE. Instead for IrO_2_, the onset potential for oxygen evolution was observed at a much higher potential of 1.48 V vs RHE. This indicates that, under the present synthesis and testing conditions, EL-4 displays more favorable OER kinetics than IrO_2_. Based on the OER oxygen current derived from EC-MS, the Tafel slope of EL-4 at small current densities (<1 mA/cm^2^) was calculated to be 38.8 mV/dec, while this value is 43.6 mV/dec for IrO_2_ (Figure 4(c)). Therefore, the exchange current density of EL-4 is 5.18 × 10^−6^ mA/cm^2^, which is 20 times higher than that of IrO_2_ (2.66 × 10^−7^ mA/cm^2^). This result again indicates the promising electrochemical properties of EL-4.

**Figure 5.**
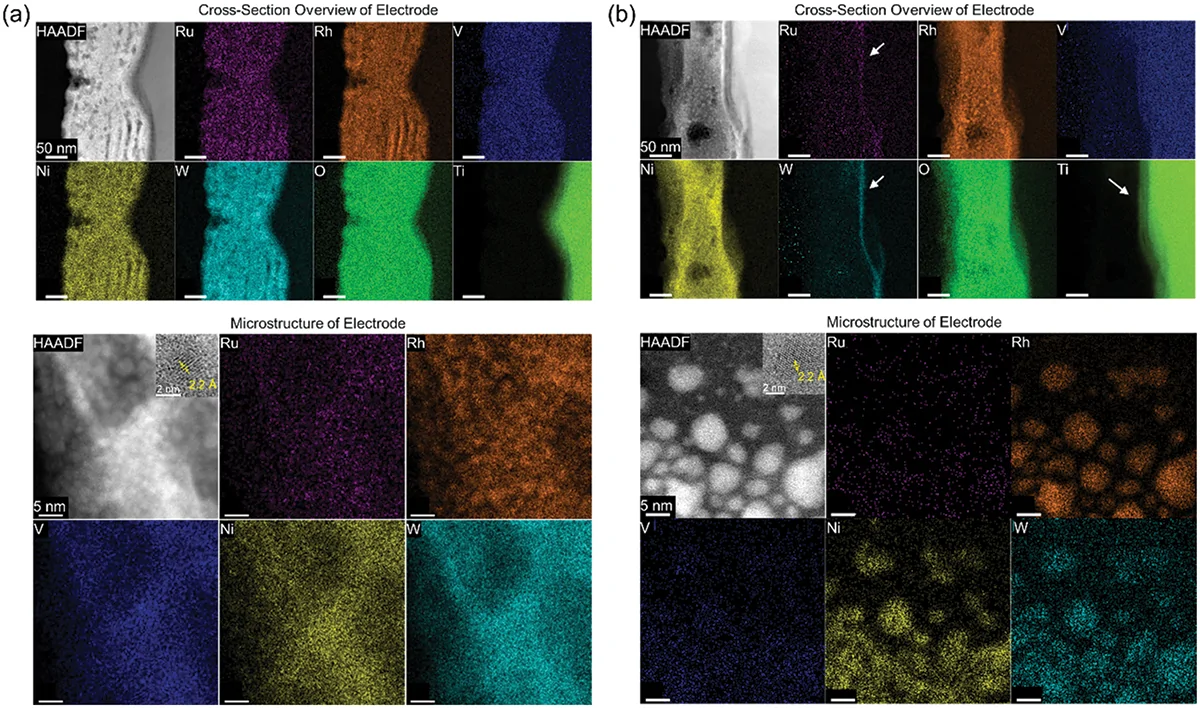
TEM observation of as-prepared (a) and activated EL-4 electrode (b). Top: cross-sectional HAADF-STEM images for overview of the electrode and the corresponding elemental mapping images. The scale bar is 50 nm. Bottom: HAADF-STEM microstructure and the corresponding elemental mapping images. The scale bar indicates 5 nm. Inset: HRTEM image of a representative single particle.

To investigate the structural origin of the high OER activity of EL-4, the microscopic morphology, element composition, and surface valence states of the as-prepared EL-4 were characterized by scanning transmission electron microscopy (STEM) and energy dispersive X-ray spectroscopy (EDS) (Figure 5(a)). The cross-sectional high-angle annular dark field STEM (HAADF-STEM) image of the as-prepared EL-4 displays that the electrocatalyst film has an average thickness of 190 nm. The corresponding EDS element mapping images reveal the homogeneous distribution of Ru, Rh, V, Ni, W and O in the sample. Further investigation at high magnification (Figure 5(a), bottom panel) demonstrates the existence of small spherical particles in diameters less than 4 nm, which are composed of Rh, Ni and O elements. The high-resolution TEM (HRTEM) image in the inset of the HAADF-STEM image shows lattice fringes of 2.2 Å. Furthermore, the X-ray diffraction (XRD) pattern of EL-4 (Figure S3) reveals a reflection at the (200) plane, matching a NiO structure (JCPDS No. 47–1049). Therefore, the as-prepared EL-4 consists of rhodium-incorporated nickel oxide embedded in the multielement amorphous metal oxide. The XPS spectra (Figure S4) further confirm the presence of Ru, Rh, V, Ni, W and O in the material, which is essentially consistent with the HAADF-STEM elemental mapping results. The Rh element exists as Rh^3+^, while the other metal elements (Ru, V, Ni, W) have the mixed valence states. In the O 1s XPS spectrum, the peaks at 530.4 and 531.8 eV correspond to lattice oxygen (metal-bonded oxygen, M-O) and hydroxide groups (−OH), respectively.

Next, the morphology and compositions of EL-4 after electrochemical activation were investigated (Figure 5(b)). As multielement electrocatalysts transform their compositions and morphologies at the early stage of electrochemical reactions and evolve into active phases, we examined this activated phase of EL-4 in order to characterize the catalytically relevant state of the material. After the electrochemical activation step, the film thickness decreased to ~160 nm, and the corresponding elemental mapping indicates the dissolution of V during the activation. As depicted in the XPS survey spectrum (Figure S5), the V 2p peak disappears after activation, indicating the absence of this element on the surface. Moreover, Ru and W are no longer uniformly distributed in the sample but rather enriched their concentrations at the interface of the sample and the substrate (Figure 5(b), top panel, indicated as white arrows). The redistribution or dissolution of elements such as V and W can introduce oxygen vacancies, which not only create faster pathways for ion diffusion but also promote favorable OH- adsorption on the surface [36–38]. Additionally, the compositional rearrangement may contribute to electrochemical reconstruction, potentially leading to transformation or exposure of more active sites. The activated EL-4 retains the NiO phase (Figure S6). As shown in the high-magnification TEM images and EDS mapping results in the bottom panel group of Figure 5(b), Rh, Ni, W and V are incorporated into larger nanoparticles, which suggests the diffusion of W and V into the rhodium nickel oxide structure during OER. The alloying of Ni, W, V and Rh modulates the local electronic structure of EL-4, thereby optimizing the adsorption energies of OER intermediates and improving electrocatalytic activities [39–42]. Figure S7 shows the high-resolution XPS spectra. The O 1s XPS spectrum is also deconvoluted into two peaks belonging to lattice oxygen and hydroxide. However, the contribution of hydroxide after the OER test greatly increased from 14% to 84%. The increase of the hydroxyl group is associated with the formation of oxygen vacancies during OER, which plays an important role in enhancing OER performance [37,43]. The transformation between the pristine material and the active phase poses a challenge for data-driven catalyst design. Traditional ML models predominantly rely on static descriptors of initial materials. However, our experimental results demonstrate that electrocatalytic performance depends on active-phase evolution under operating conditions. Therefore, integrating parameters that capture electrochemical reconstruction as dynamic ML descriptors is vital for accurately predicting true electrocatalytic performance.

**Figure 6.**
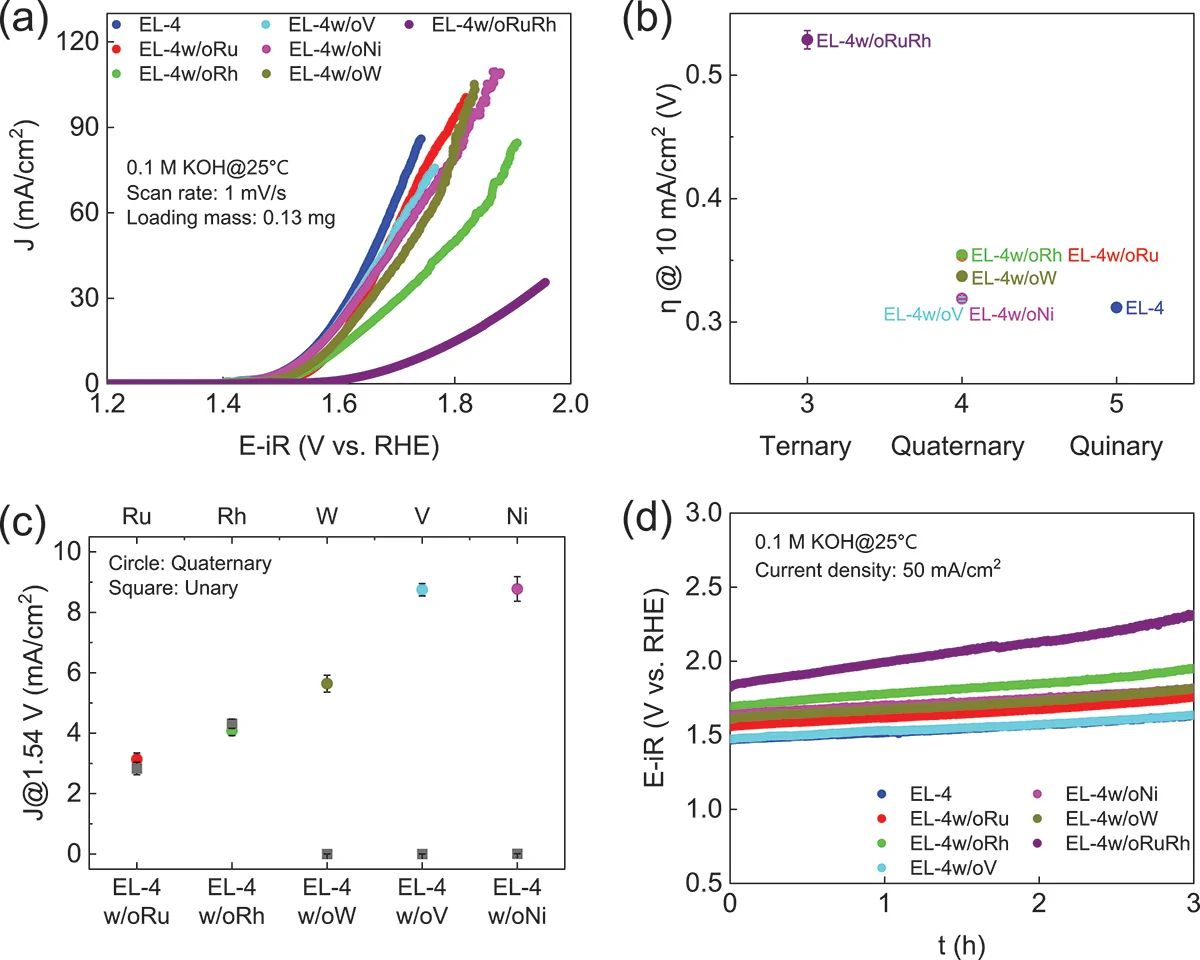
OER performances of multielement electrocatalysts measured in 0.1 M KOH electrolyte. (a) LSV curves measured at a scan rate of 1 mV/s. (b) Overpotentials at a current density of 10 mA/cm^2^. (c) Current densities at 1.54 V vs RHE. (d) CP curves at a current density of 50 mA/cm^2^ for 3 h.

To reveal the effect of metal elements on OER activity, we further prepared corresponding quaternary-metal catalysts EL-4w/o M, where M denotes the absent metal element from EL-4 and checked their electrochemical properties. As shown in Figure 6(a), all the quaternary electrocatalysts show the worse OER performance compared to the quinary EL-4. When Rh or Ru is unavailable, the OER activity is significantly decreased. The overpotential values at 10 mA/cm^2^ for EL-4w/oRh (= Ru_0.5_V_0.5_Ni_0.5_W) and EL-4w/oRu (= Rh_0.5_V_0.5_Ni_0.5_W) are 354.0 and 351.5 mV, respectively, which result shows an increase of more than 36 mV in comparison to EL-4 (Figure 6(b)). We put an emphasis here that EL-4w/oRh shows much lower current densities at high overpotentials than the other quaternary catalysts. Clearly, the OER activities of the quaternary compounds are inversely correlated with those of the unary ones (Figure S8). Their current densities at 1.54 V vs RHE are displayed in Figure 6(c). There is an opposite trend on current densities for unary oxides and their corresponding remaining-4-element oxides. When both Rh and Ru were removed, a ternary catalyst EL-4w/oRuRh (= V_0.5_Ni_0.5_W) was obtained. The electrocatalytic activity of EL-4w/oRuRh is further degraded, leading to a rather high overpotential of 520.8 mV at 10 mA/cm^2^. The chronopotentiometric results (Figure 6(d)) also show that EL-4w/oRuRh is not as stable as the other quaternary and quinary materials. Based on the above results, the quinary electrocatalysts achieved enhanced OER activity and durability compared to quaternary and ternary materials. The excellent electrocatalytic performance of the quinary material should be attributed to the synergistic effect between multiple elements [44–46].

## Conclusions

In conclusion, an interpretable data-driven framework using a small hybrid dataset has been developed for the extrapolative discovery of high-performance multielement oxygen evolution electrocatalysts from a large material search space, based on a small hybrid database composed of literature and experimental data. Our results identified a promising quinary oxide with a metal composition of Ru:Rh:V:Ni:W = 0.5:0.5:0.5:0.5:1. Electrochemical experiments demonstrated that this quinary oxide achieved a lower overpotential at 10 mA/cm^2^ and showed improved alkaline oxygen evolution activity relative to IrO_2_ and other oxides synthesized under the same condition. The consistency of experiments and predictions confirmed the effectiveness of our small-data-driven approach. Through further experiments, the quinary catalyst has been proved to exhibit higher OER activity than the oxides containing combinations of less metal elements due to multi-element synergistic effect. This highlights the necessity of the interpretable data-driven approach for the accelerated discovery of multielement metal oxide electrocatalysts and their understanding for better electrochemical properties.

## Experimental

### Feature calculation

To represent the composition of the electrocatalysts, 17 group coefficients and 17 group weighted properties (atomic radius in this case) were calculated as follows.

The group coefficient is derived from the total magnitude of the coefficient of a given element in the chemical formula of the electrocatalyst, if more than one element in the formula belongs to the same group, they are added. For example, in the formula Ba_0.5_Co_0.5_Fe_1._5O_5_PrSr0._5_ group coefficient 2 would equal 1 (Ba + Sr), while group coefficients 3, 8 and 9 would be 1 (Pr), 1.5 (Fe) and 0.5 (Co), respectively. For this study, all electrocatalysts in the curated data were deemed to be oxides, so oxygen was not counted in the calculation of group coefficient 16; thus it was deemed 0.

The weighted atomic radius was input as equal to the value of the atomic radius of the element in picometers. If more than one element belongs to the same group (such as Ba and Sr in the above formula) its value is calculated as a weighted average of them. Consequently, the weighted atomic radius for Ba_0.5_Co_0.5_Fe_1._5O_5_PrSr0._5_ would be represented as 236 for group 2 (Ba + Sr, 0.5 × 253 + 0.5 × 219), and for groups 3, 8 and 9 it would be represented as 247 (Pr), 156 (Fe) and 152 (Co), if the electrocatalyst contains only oxygen for group 16, the atomic radius value was deemed 0.

### Chemicals and materials

Nickel chloride (NiCl_2_·6 H_2_O, 98.0%), ruthenium chloride (RuCl_3_, 99.9%), oxalic acid (H_2_C_2_O_4_, 99%) and ethanol (99.5%) were purchased from FUJIFILM Wako, Japan. Tungsten chloride (WCl_6_, ≥99.9%), iridium chloride (IrCl_3,_ 99.9%), rhodium chloride (RhCl_3_, 99.95%), osmium chloride (OsCl_3_, 99.9%), niobium chloride (NbCl_5_, 99.995%) and potassium hydroxide (KOH, 99.99%) were obtained from Sigma-Aldrich, U.S.A. Vanadium chloride (VCl_3_, min 95%) was purchased from Strem Chemicals, Inc., U.S.A., palladium chloride (PdCl_2_, 99.999%) from Alfa Aesar, U.S.A., and acetone (99.5%) from KANTO KAGAKU, Japan. Milli-Q water (Merck KGaA, Germany; 18.2 MΩ·cm, TOC < 5 ppb) was used for preparing ultrapure electrolyte solutions. Titanium foil (Ti, 99.5%) was purchased from Nilaco, Japan. The foil was cut into 0.05 mm × 5 mm × 30 mm, cleaned by acetone, and then etched in H_2_C_2_O_4_ aqueous solution (10%) at 80°C for 1 h. After washing with deionized water and dried, the etched Ti was used as substrate for electrocatalyst deposition.

### Electrocatalyst synthesis

Electrocatalysts were synthesized using a dip-coating method described as the following. First, 0.1 M metal precursor solutions were prepared by dissolving metal salts in ethanol. A 0.05 M mixture was obtained by mixing the respective metal precursor solutions at a desired ratio, e.g. Ru : Rh : V : Ni : W = 0.5 : 0.5 : 0.5 : 0.5 : 1 for EL-4 (Ru_0.5_Rh_0.5_V_0.5_Ni_0.5_W). Then the mixed solution (2.5 μL) was dropped on an etched Ti substrate. A thin film was formed after drying at room temperature followed by heating at 350°C for 10 min. The deposition and heating process was performed successively for 10 times. Finally, the film was calcined at 450°C for 1 h, resulting in an oxide layer on the Ti substrate. For performance comparison, an IrO_2_ film was also prepared using the same procedure.

### Electrochemical measurements

The electrochemical measurements were performed on a multichannel potentio/galvanostat (VMP-3, Bio-Logic, France) in 0.1 M KOH aqueous solution at room temperature. In a standard three-electrode cell configuration, the as-prepared thin-film electrocatalysts were directly employed as the working electrodes. A platinum wire and a Ag/AgCl electrode were used as the counter electrode and the reference electrode, respectively. The polarization curves were obtained by linear sweep voltammetry (LSV) at a scan rate of 1 mV/s, and the durability test was carried out by chronopotentiometry under a current density of 50 mA/cm^2^. Cyclic voltammetry (CV) was measured between 0.6–0.8 V vs RHE at different scan rates of 5, 10, 20, 30 and 40 mV/s.

### Electrochemical-mass spectroscopy measurements

Oxygen evolution was monitored on an electrochemistry mass spectrometry (EC-MS) system (Spectro Inlets ApS, Denmark). In this system, an electrochemical (EC) cell is coupled with a mass spectrometer (MS) by a membrane chip. The EC cell was installed, with a platinum disk coated with electrocatalysts as the working electrode, a platinum wire as the counter electrode and an Ag/AgCl electrode as the reference electrode, respectively. Measurements were carried out in 0.1 M KOH electrolyte, with a continuous flow of argon as carrier gas. EC data were obtained on VMP-3 and MS oxygen signals were collected using Zilien software.

### Material characterization

X-ray diffraction (XRD) patterns were recorded on an X-ray diffractometer (Rigaku, Miniflex 600, Japan) with Cu Kα radiation. Cross-sectional morphologies were observed using transmission electron microscopy/ scanning transmission electron microscopy (TFS Spectra Ultra TEM/STEM, Thermo Fisher Scientific, USA) operated at 300 kV, equipped with an energy-dispersive X-ray spectroscopy (EDS) detector for element analysis. Before observation, the sample was cut by focused ion beam (JIB-4000). X-ray photoelectron spectrometry (XPS) was carried out using an X-ray photoelectron spectrometer (PHI Quantes, ULVAC-PHI, Japan) with a monochromatic Al Kα X-ray source (1486.6 eV). The obtained XPS spectra were calibrated using the C1s peak located at 284.8 eV.

## Supplementary Material

Supplemental Material
