# Supplementary material for: Interpretable data-driven discovery of high-performance multielement metal oxide electrocatalysts for oxygen evolution reaction from a small hybrid dataset
Source: Sci Technol Adv Mater. 2026 Aug 7;27(1):2713992. doi: 10.1080/14686996.2026.2713992 (PMC13495041; doi:10.1080/14686996.2026.2713992)
Supplement: Supplementary file 1 [file TSTA_A_2713992_SM7509.docx]

Supporting Information

**Interpretable Data-Driven Discovery of High-Performance Multielement Metal Oxide Electrocatalysts for Oxygen Evolution Reaction from a Small Hybrid Dataset**

*Wenqin Peng,^a^ Shigenobu Hayashi,^b^ Abraham Castro Garcia,^a^ Lei Fang,^b^ Masaki Takeguchi,^a^ Yibin Xu^b^ and Ken Sakaushi^a,c,d^**

^a^Research Center for Energy and Environmental Materials, National Institute for Materials Science, Namiki 1-1, Tsukuba 305-0044, Japan

^b^Center for Basic Research on Materials, National Institute for Materials Science, Sengen 1-2-1, Tsukuba, Ibaraki, 305-0047, Japan

^c^Churchill College, Cambridge, United Kingdom

^d^Cavendish Laboratory, University of Cambridge, Cambridge, United Kingdom

Table S1. Metal-element composition and ratio of the five top quinary-metal oxide-based electrocatalysts predicted by small-data-driven artificial intelligence.

Table S2. Metal-element composition and ratio of quaternary and ternary electrocatalysts derived from EL-4.

Figure S1. OER polarization curves normalized by mass loading in 0.1 M KOH.

Figure S2. CP curves at a current density of 50 mA/cm^2^ for 3 h.

Figure S3. XRD of the electrocatalysts and Ti substrate.

Figure S4. High-resolution XPS spectra of the as-prepared EL-4

Figure S5. XPS survey spectra of EL-4 before and after OER testing.

Figure S6. XRD of EL-4 before and after OER testing.

Figure S7. High-resolution XPS spectra of EL-4 after OER testing.

Figure S8. LSV curves of unary oxide in 0.1 M KOH at a scan rate of 1 mV/sec.
